# Lipids, apolipoproteins and gestational diabetes mellitus: a Mendelian randomization study

**DOI:** 10.1186/s12884-024-06556-2

**Published:** 2024-05-06

**Authors:** Dan Shan, Ao Wang, Ke Yi

**Affiliations:** Key Laboratory of Obstetrics and Gynecologic and Pediatric Diseases and Birth Defects of Ministry of Education, West China Second University Hospital, Sichuan University, Chengdu, 610041 China

**Keywords:** Lipids, Apolipoproteins, Gestational diabetes mellitus, Mendelian randomization

## Abstract

**Background:**

This study investigates the causal relationship between lipid traits and GDM in an effort to better understand the aetiology of GDM.

**Methods:**

Employing a two-sample Mendelian Randomization (MR) framework, we used Single Nucleotide Polymorphisms (SNPs) as instrumental variables to examine the impact of lipids and apolipoproteins on GDM. The research comprised univariable and multivariable MR analyses, with a prime focus on individual and combined effects of lipid-related traits. Statistical techniques included the fixed-effect inverse variance weighted (IVW) method and supplementary methods such as MR-Egger for comprehensive assessment.

**Results:**

Our findings revealed the following significant associations: apoA-I and HDL cholesterol were inversely correlated with GDM risk, while triglycerides showed a positive correlation. In multivariable analysis, apoA-I consistently exhibited a strong causal link with GDM, even after adjusting for other lipids and Body Mass Index (BMI).

**Conclusion:**

The study demonstrates a significant causal relationship between apoA-I and GDM risk.

**Supplementary Information:**

The online version contains supplementary material available at 10.1186/s12884-024-06556-2.

## Introduction

Gestational Diabetes Mellitus (GDM) represents a major public health concern due to its increasing prevalence and profound effects on both maternal and foetal health [1, 2]. Approximately 5–7% of pregnancies are estimated to be impacted by GDM, with variations depending on the population studied and diagnostic standards [3]. Characterised by glucose intolerance first identified during pregnancy, GDM is linked to an elevated risk of various adverse outcomes [4]. These include a higher likelihood of cesarean delivery, pre-eclampsia, and the development of type 2 diabetes in later life for mothers [5–7]. For infants, the risks extend to macrosomia, hypoglycaemia, and a predisposition to obesity [8, 9].

Effective strategies for prevention, early detection, as well as management of GDM can mitigate short-term complications and offer a chance to improve long-term health outcomes [10, 11]. This underscores the need for continued research into its pathophysiology, risk factors, and effective interventions. Environmental factors, lifestyle choices, and genetics all have a role in the pathophysiology of GDM [12, 13]. Research into the role of lipid metabolism in GDM highlights its significance in the pathogenesis of this condition. Observational studies have demonstrated that dysregulated lipid profiles, including elevated triglycerides and low HDL cholesterol levels, are commonly observed in GDM. These lipid imbalances contribute to insulin resistance, a hallmark of GDM [14]. Additionally, a lot of attention has been given to the role of specific apolipoproteins, particularly Apolipoprotein A-I (apoA-I) and Apolipoprotein B(apoB), in modulating lipid metabolism and influencing GDM risk. Wu et al. found that apoA-I protects rats from pregnancy-induced insulin resistance by increasing insulin sensitivity and inhibiting inflammation in adipose tissue and skeletal muscle [15]. Zheng et al. reported that the serum levels of triglycerides, LDL cholesterol, and Apolipoprotein B during the first trimester of pregnancy have important clinical value in predicting GDM [16]. However, the causal nature of this association is yet unclear and requires further investigation.

Mendelian Randomization (MR) is a method that leverages genetic variations as tools to infer causal relationships between risk factors and diseases [17]. In MR studies, genetic variants known to affect lipid levels (such as those affecting HDL cholesterol, LDL cholesterol, and triglyceride levels) are employed as instrumental variables. These variants are generally unaltered by environmental factors and disease states, making them ideal for examining the causal effect of lipid levels on GDM risk. This robust methodology may provide valuable insights into the underlying mechanisms while shedding light on the biological pathways linking lipid-related traits to GDM.

## Materials and methods

### Study design

**Fig. 1:**
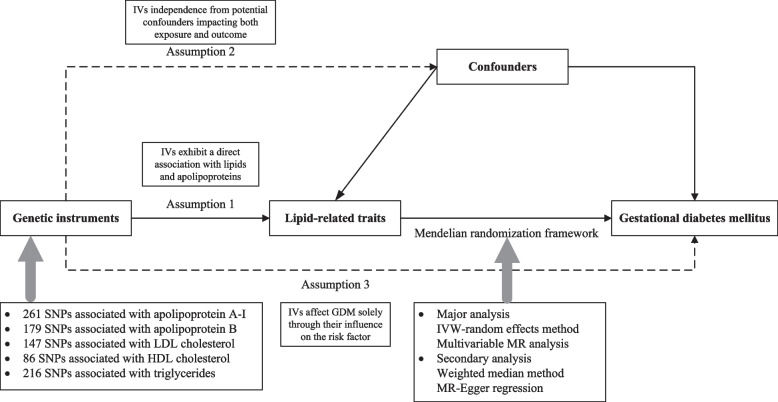
Overview of the MR analysis process. Abbreviations: MR, mendelian randomization; IVs, instrumental variables; IVW, Inverse variance weighted; HDL-C, High density lipoprotein cholesterol; LDL-C, Low density lipoprotein cholesterol

In this research, we conducted a two-sample Mendelian randomization (MR) analysis in order to assess the causal link between lipids and apolipoproteins and GDM. SNPs served as instrumental variables (IVs) [18]. To enhance result accuracy, validating three key hypotheses throughout the entire process is crucial [19]. We identified genetic variants significantly associated with lipid levels and calculated the corresponding F-statistics to assess the strength of each variant as an instrumental variable. We conducted an analysis of confounding factors to ensure that the selected variants are not associated with known confounders, such as BMI. We also used methods such as MR-Egger regression to evaluate the potential pleiotropy of the genetic variants, further confirming that their effects on GDM are primarily mediated through lipid levels (Fig. 1).

The univariable MR analysis sought to analyse the correlation between specific lipid-related traits and GDM. The multivariable MR analysis, on the other hand, aimed to assess the individual impacts of interrelated lipid-related traits on GDM [20]. Both analyses aimed to comprehend the relationship between lipid-related traits and the risk of GDM, with the univariable focusing on individual traits and the multivariable concentrating on their interactions. All original studies obtained ethical review approval and informed consent. Genetic instruments for apoA-I, apoB, LDL cholesterol, HDL cholesterol, and triglycerides were extracted from the IEU Open GWAS database (Supplementary Table S1).

### Statistical analyses

Our main approach for MR analysis was the fixed-effect inverse variance weighted (IVW) method. In cases where potential heterogeneity among selected SNPs was present, random effects modelling was employed [21]. Additionally, we utilised four other effective methods—MR-Egger, weighted median, weighted mode, and simple mode—to comprehensively analyse the potential relationship. It is noteworthy that although these methods offer a comprehensive evaluation, they might have less statistical power compared to the IVW test. We employed Cochran’s Q statistic and the MR-Egger test for assessing heterogeneity and pleiotropy, respectively.

### Genetic instrument selection

In univariable MR analysis, independent SNPs linked to apoA-I, apoB, LDL cholesterol, HDL cholesterol, and triglycerides were isolated using a threshold of linkage disequilibrium clumping (r^2^ = 0.001) and a window size of 10 megabases. Specifically, we focused on genome-wide significant SNPs (*p* < 5e-8) associated with each trait so as to reduce redundancy.

### Sensitivity analyses

To ensure the reliability of the identified causal effect of lipids and apolipoproteins on GDM, we carried out a thorough set of sensitivity analyses. Cochran’s Q statistic was utilised to assess potential heterogeneity within the data [22]. The MR-Egger intercept analysis was employed to investigate horizontal pleiotropy [23]. We also conducted a Leave-one-out analysis to examine if any single SNP substantially affected the outcomes by systematically removing SNPs individually. Additionally, reverse MR analyses were performed to explore the potential reverse causal link between lipids and apolipoproteins (as seen in the forward MR analysis) and GDM.

For multivariable MR analysis, we applied two models to further understand the connection between lipid-related traits and GDM risk. In Model 1, five lipid-related traits (apoA-I, apoB, LDL cholesterol, HDL cholesterol, and triglycerides) were included in multivariable analysis.

In Model 2, we included BMI for analysis, along with the three traits that showed positive associations in univariable analysis: apoA-I, HDL cholesterol, and triglycerides.

All analyses were performed using R (version 4.2.0) and RStudio, employing the R packages “TwoSampleMR” and “MR-PRESSO”.

## Results

### Univariable Mendelian randomization analysis

After excluding SNPs associated with confounders, we identified 261 instrumental variables for apoA-I, 179 IVs for apoB, 86 IVs for HDL cholesterol, 147 IVs for LDL cholesterol, and 216 IVs for triglycerides. F-statistics of Instrument Variables for lipids and apolipoproteins are shown in Supplementary Table S7.

**Fig. 2:**
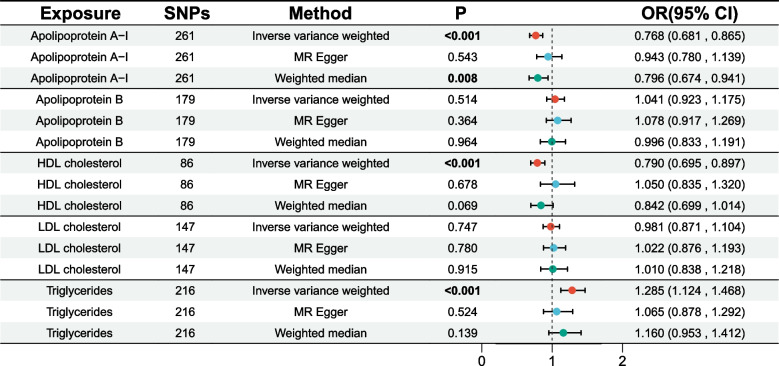
Univariable Mendelian randomization results using different methods. Abbreviations: SNP, Single nucleotide polymorphism; HDL-C, High density lipoprotein cholesterol; LDL-C, Low density lipoprotein cholesterol; OR, Odds ration; CI, Confidence interval

A significant correlation between apoA-I and the risk of GDM was determined through the IVW technique (OR [95%CI] = 0.76 [0.68–0.86]; *p* < 0.001). Moreover, HDL cholesterol was found to be significantly associated with a lower risk of GDM (OR [95%CI] = 0.79[0.69–0.89]; *p* < 0.001). Triglycerides were found to be significantly linked to an elevated risk of GDM (OR [95%CI] = 1.28[1.12–1.46]; *p* < 0.001). (Fig. 2 and Supplementary Table S3).

A reverse MR analysis was conducted to explore the potential causal effect of GDM on lipid-related traits. The findings suggested no reverse causal relationship between GDM and each trait (Supplementary Table S4).

### Multivariable Mendelian randomization analysis

**Fig. 3:**
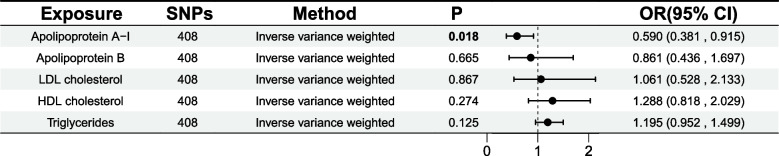
Multivariable Mendelian randomization using the inverse-variance weighted method in model 1. Model 1 included Apolipoprotein A-I, Apolipoprotein B, LDL cholesterol, HDL cholesterol and triglycerides. Abbreviations: SNP, Single nucleotide polymorphism; HDL-C, High density lipoprotein cholesterol; LDL-C, Low density lipoprotein cholesterol; OR, Odds ration; CI, Confidence interval

Figure 3 presents the outcomes of the multivariable MR analysis in model 1. When adjusting simultaneously for apoA-I, apoB, LDL cholesterol, HDL cholesterol, and triglycerides, apoA-I continued to have a strong causal link with GDM; the OR was 0.59 (95% CI = 0.38, 0.91). However, the effects for HDL cholesterol and triglycerides were greatly reduced (Supplementary Table S5).

**Fig. 4:**
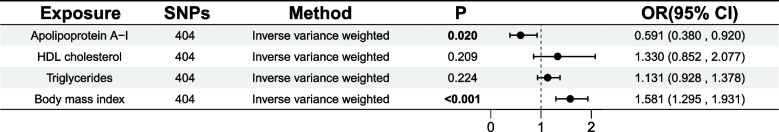
Multivariable Mendelian randomization using the inverse-variance weighted method in model 2. Model 2 included Apolipoprotein A-I, HDL cholesterol, triglycerides and Body mass index. Abbreviations: SNP, Single nucleotide polymorphism; HDL-C, High density lipoprotein cholesterol; OR, Odds ration; CI, Confidence interval

Figure 4 exhibits the outcomes of the multivariable MR analysis in model 2. Body mass index is known as a risk factor for GDM. For model 2, the subjects included the three traits with positive results in univariable analysis (apoA-I, HDL cholesterol, and triglycerides) and BMI. When adjusting simultaneously for apoA-I, HDL cholesterol and triglycerides, and BMI, apoA-I consistently showed a strong causal association with GDM; the OR was 0.59 (95% CI = 0.38, 0.92). However, the estimates of HDL cholesterol and triglycerides were significantly reduced (Supplementary Table S6).

### Sensitivity analysis

In our analysis of apoB and HDL cholesterol causal impacts on GDM, instrumental heterogeneity was detected (Cochran’s Q test, *p* < 0.05; Supplementary Table S2), leading us to employ the random-effects IVW method. On the other hand, for other analyses where no heterogeneity was observed (Cochran’s Q test, *p* > 0.05), the fixed-effects IVW method was applied.

There was no evidence of horizontal pleiotropy in the MR-Egger intercept analysis results. Scatter plots illustrated the causal effect of lipid-related traits on GDM across the five MR methods; a positive relationship is indicated by a slope greater than zero, and vice versa (Supplementary Figure S1). Furthermore, no discernible heterogeneity was shown by the Funnel plot symmetry (Supplementary Figure S2).

## Discussion

The incidence of gestational diabetes mellitus (GDM) is increasing worldwide and poses a major concern for the health of pregnant women and their fetuses [24, 25]. Our comprehensive investigation into the role of lipids and apolipoproteins in GDM is essential because they play a key role in metabolic pathways that may have an important impact on pregnancy outcomes [26].

Our study explored the intricate interplay between lipids, apolipoproteins, and GDM. ApoA-I is the major protein component of HDL and plays a critical role in reverse cholesterol transport, a key process in removing cholesterol from tissues and returning it to the liver for excretion. Conversely, apoB is a primary component of LDL, very-low-density lipoprotein, and intermediate-density lipoprotein, which are involved in the transport of cholesterol and triglycerides from the liver to peripheral tissues.

The noteworthy associations revealed between these biomarkers and GDM provide novel insights into their potential roles in the pathogenesis of this condition. In the univariable Mendelian randomization analysis, compelling associations were discovered between lipid and apolipoprotein levels and the risk of GDM. Importantly, apoA-I has demonstrated an inverse correlation with GDM risk, suggesting its potential protective role. This is consistent with the established function of apoA-I in facilitating reverse cholesterol transport and its anti-inflammatory properties, which could potentially mitigate GDM risk through enhanced lipid metabolism as well as reduced inflammation [27, 28]. Similarly, the inverse association between HDL cholesterol and the risk of GDM is indicative of the protective role of high-density lipoproteins in cardiovascular health, potentially exerting a similar influence on GDM by modulating lipid homeostasis and insulin sensitivity [29, 30]. On the other hand, dysregulated triglyceride levels may increase vulnerability to GDM, as suggested by the positive connection found between triglycerides and GDM risk. This relationship highlights the effect of high triglyceride levels on insulin resistance and impaired glucose metabolism.

In multivariable Mendelian randomization analyses, two distinct models provided intriguing insights into the relationship between lipid profiles and gestational diabetes mellitus (GDM). Model 1, which encompassed adjustments for all pertinent lipid and apolipoprotein features, notably highlighted apoA-I’s sustained significant association with GDM. This reinforces the robustness of apoA-I’s impact on GDM risk independent of other lipid factors. Interestingly, although there were initial significant correlations between HDL cholesterol and triglycerides in the univariable analysis, their effects diminished in Model 1, suggesting a potential attenuation or mediation of their individual associations with GDM when adjusting for other lipid factors.

The critical role of apoA-I in GDM was further highlighted in Model 2 by the inclusion of BMI. Even after adjusting for BMI, apoA-I maintained a robust association with GDM, emphasising its independent contribution to GDM risk [31]. However, the effects of HDL cholesterol and triglycerides were notably attenuated in this adjusted model, suggesting a potential interplay between these lipid traits and BMI in influencing GDM susceptibility. These findings underscore apoA-I’s consistent and considerable relationship with GDM, irrespective of BMI adjustments, while also pointing to the need for deeper exploration into the complex interrelationships among lipids, BMI, and GDM susceptibility to gain a more comprehensive understanding of their collective impact.

Our study has identified a robust causal association between apoA-I and GDM, wherein elevated levels of apoA-I correspond to a significant reduction in GDM risk. This is partly in line with previous research. Metformin is a widely used insulin sensitizer [32]. As claimed by Karavia et al., the sensitizing effect of metformin is diminished in mice with apoA-I gene knock-down (apoA-I (-/-)), revealing that apoA-I may be involved in insulin sensitization [33]. A cross-sectional study found that low apoA-I was associated with insulin resistance in patients with impaired glucose tolerance [27]. However, Retnakaran et al. found no significant association between serum apoa-1 levels and the risk of insulin resistance or GDM in pregnant women in an observational study [34]. This discrepancy may be attributed to variations in study design and methodology, underlining the complexity involved in determining the precise role of apoA-I in GDM pathogenesis.

Our study uncovers a potential causal relationship between apoA-I levels and the risk of gestational diabetes, which could facilitate early prediction of GDM, inform prevention strategies and treatment interventions, and promote the advancement of personalized medicine.

It is important to note that our study has a number of limitations. Firstly, MR studies rely on certain assumptions, such as the absence of pleiotropy and horizontal pleiotropy, which could have an effect on the validity of the causal inference. While employing robust genetic instruments and sensitivity analyses to mitigate these concerns, complete elimination of residual confounding remains challenging. Secondly, our research also concentrated on the genetic effects of lipid-related traits on GDM risk. Although we adjusted for BMI in multivariable MR analysis, other factors, including environmental and lifestyle factors, were not taken into account. Subsequent studies should strive to incorporate these elements into their analyses, contributing to a more holistic comprehension of the causal mechanisms underlying the relationship between lipid-related traits and GDM. Thirdly, the summary statistics used in our study encompass data from both male and female participants and do not distinguish between lipid levels or BMI measured before and after pregnancy. This limitation may impact the specificity of our findings related to the risk of GDM, as the physiological conditions of these distinct groups can differ substantially. Additionally, a significant limitation of this study is the reliance on summary statistics, which restricts our ability to investigate non-linear relationships between lipid levels and the risk of GDM. The analysis operates under the assumption that these relationships are linear, which may not adequately capture the complexities inherent in lipid metabolism. This methodological simplification might fail to detect clinically significant non-linear effects, indicating that future research would benefit from employing more sophisticated methods capable of exploring these dynamics in greater detail.

In conclusion, our study strongly suggests a potential causal relationship between genetic susceptibility to apoA-I and a reduced risk of GDM. Further validation of our findings and investigation into the underlying biological mechanisms warrant additional research, which may advance personalised approaches to GDM prevention and management.

### Supplementary Information

Supplementary Material 1.

## Data Availability

Original data generated and analyzed during this study are included in this published article or supplementary material.

## Abbreviations

GDM: Gestational Diabetes Mellitus
apoA-I: Apolipoprotein A-I
apoB: Apolipoprotein B
HDL-C: High-density lipoprotein cholesterol
LDL-C: Low-density lipoprotein cholesterol
BMI: Body mass index
GWAS: Genome-wide association study
MR: Mendelian randomization
SNP: Single nucleotide polymorphism
IV: Instrumental variable
IVW: Inverse variance weighted
MR-PRESSO: Mendelian randomization pleiotropy residual sum and outlier
LD: Linkage disequilibrium
OR: Odds ration
CI: Confidence interval
HDL: High density lipoprotein
LDL: Low-density lipoprotein

## References

[1] Johns EC, Denison FC, Norman JE, Reynolds RM (2018). Gestational diabetes mellitus: mechanisms, treatment, and complications. Trends Endocrinol Metab.

[2] McIntyre HD, Catalano P, Zhang C, Desoye G, Mathiesen ER, Damm P (2019). Gestational diabetes mellitus. Nat Rev Dis Primers.

[3] Sweeting A, Wong J, Murphy HR, Ross GP (2022). A clinical update on gestational diabetes mellitus. Endocr Rev.

[4] Choudhury AA, Devi RV (2021). Gestational diabetes mellitus - a metabolic and reproductive disorder. Biomed Pharmacother.

[5] Buchanan TA, Xiang AH, Page KA (2012). Gestational diabetes mellitus: risks and management during and after pregnancy. Nat Rev Endocrinol.

[6] Kim C (2014). Maternal outcomes and follow-up after gestational diabetes mellitus. Diabet Med.

[7] Yang Y, Wu N (2022). Gestational diabetes mellitus and preeclampsia: correlation and influencing factors. Front Cardiovasc Med.

[8] Monteiro LJ, Norman JE, Rice GE, Illanes SE (2016). Fetal programming and gestational diabetes mellitus. Placenta.

[9] Kautzky-Willer A, Harreiter J, Winhofer-Stöckl Y, Bancher-Todesca D, Berger A, Repa A (2019). Gestational diabetes mellitus (Update 2019). Wien Klin Wochenschr.

[10] Kintiraki E, Goulis DG (2018). Gestational diabetes mellitus: Multi-disciplinary treatment approaches. Metabolism.

[11] Simmons D, Immanuel J, Hague WM, Teede H, Nolan CJ, Peek MJ (2023). Treatment of gestational diabetes mellitus diagnosed early in pregnancy. N Engl J Med.

[12] Plows JF, Stanley JL, Baker PN, Reynolds CM, Vickers MH (2018). The pathophysiology of gestational diabetes mellitus. Int J Mol Sci.

[13] Hu G, Liu H, Leng J, Wang L, Li W, Zhang S (2022). Effects of a lifestyle intervention in young women with GDM and subsequent diabetes. Nutrients.

[14] Layton J, Powe C, Allard C, Battista MC, Doyon M, Bouchard L (2019). Maternal lipid profile differs by gestational diabetes physiologic subtype. Metabolism.

[15] Wu BJ, Sun Y, Ong KL, Li Y, Tang S, Barter PJ (2019). Apolipoprotein A-I protects against pregnancy-induced insulin resistance in rats. Arterioscler Thromb Vasc Biol.

[16] Zheng Y, Hou W, Xiao J, Huang H, Quan W, Chen Y (2022). Application value of predictive model based on maternal coagulation function and glycolipid metabolism indicators in early diagnosis of gestational diabetes mellitus. Front Public Health.

[17] Emdin CA, Khera AV, Kathiresan S (2017). Mendelian randomization. JAMA.

[18] Lawlor DA, Harbord RM, Sterne JA, Timpson N, Davey SG (2008). Mendelian randomization: using genes as instruments for making causal inferences in epidemiology. Stat Med.

[19] Davies NM, Holmes MV, Davey SG (2018). Reading Mendelian randomisation studies: a guide, glossary, and checklist for clinicians. BMJ.

[20] Sanderson E (2021). Multivariable Mendelian randomization and mediation. Cold Spring Harb Perspect Med.

[21] Burgess S, Dudbridge F, Thompson SG (2016). Combining information on multiple instrumental variables in Mendelian randomization: comparison of allele score and summarized data methods. Stat Med.

[22] Bowden J, Del Greco MF, Minelli C, Zhao Q, Lawlor DA, Sheehan NA (2019). Improving the accuracy of two-sample summary-data Mendelian randomization: moving beyond the NOME assumption. Int J Epidemiol.

[23] Bowden J, Davey Smith G, Burgess S (2015). Mendelian randomization with invalid instruments: effect estimation and bias detection through Egger regression. Int J Epidemiol.

[24] Vince K, Perković P, Matijević R (2020). What is known and what remains unresolved regarding gestational diabetes mellitus (GDM). J Perinat Med.

[25] Giuliani C, Sciacca L, Biase ND, Tumminia A, Milluzzo A, Faggiano A (2022). Gestational diabetes mellitus pregnancy by pregnancy: early, late and nonrecurrent GDM. Diabetes Res Clin Pract.

[26] O'Malley EG, Reynolds CME, Killalea A, O'Kelly R, Sheehan SR, Turner MJ (2020). Maternal obesity and dyslipidemia associated with gestational diabetes mellitus (GDM). Eur J Obstet Gynecol Reprod Biol.

[27] Feng X, Gao X, Yao Z, Xu Y (2017). Low apoA-I is associated with insulin resistance in patients with impaired glucose tolerance: a cross-sectional study. Lipids Health Dis.

[28] Tang S, Tabet F, Cochran BJ, Cuesta Torres LF, Wu BJ, Barter PJ (2019). Apolipoprotein A-I enhances insulin-dependent and insulin-independent glucose uptake by skeletal muscle. Sci Rep.

[29] O'Mara AE, Johnson JW, Linderman JD, Brychta RJ, McGehee S, Fletcher LA (2020). Chronic mirabegron treatment increases human brown fat, HDL cholesterol, and insulin sensitivity. J Clin Invest.

[30] Chung ST, Katz LEL, Stettler-Davis N, Shults J, Sherman A, Ha J (2022). The relationship between lipoproteins and insulin sensitivity in youth with obesity and abnormal glucose tolerance. J Clin Endocrinol Metab.

[31] King TW, Cochran BJ, Rye KA (2023). ApoA-I and diabetes. Arterioscler Thromb Vasc Biol.

[32] Madiraju AK, Qiu Y, Perry RJ, Rahimi Y, Zhang XM, Zhang D (2018). Metformin inhibits gluconeogenesis via a redox-dependent mechanism in vivo. Nat Med.

[33] Karavia EA, Hatziri A, Kalogeropoulou C, Papachristou NI, Xepapadaki E, Constantinou C (2015). Deficiency in apolipoprotein A-I ablates the pharmacological effects of metformin on plasma glucose homeostasis and hepatic lipid deposition. Eur J Pharmacol.

[34] Retnakaran R, Ye C, Connelly PW, Hanley AJ, Sermer M, Zinman B (2019). Serum apoA1 (Apolipoprotein A-1), insulin resistance, and the risk of gestational diabetes mellitus in human pregnancy-brief report. Arterioscler Thromb Vasc Biol.

