# Supplementary material for: Lipids, apolipoproteins and gestational diabetes mellitus: a Mendelian randomization study
Source: BMC Pregnancy Childbirth. 2024 May 6;24:347. doi: 10.1186/s12884-024-06556-2 (PMC11071177; doi:10.1186/s12884-024-06556-2)

**Supplementary Content**

**Supplementary Tables.**

Table S1 . Additional information on GWAS, including covariates adjusted for.

Table S2 . Cochran Q test result and MR-Egger intercept in univariable MR.

Table S3 . Results of univariable MR.

Table S4. Effects of genetic liability to gestational diabetes mellitus on lipids and apolipoproteins in reverse Mendelian randomization analysis.

Table S5. Multivariable MR model 1 estimates for Apolipoprotein A-I, Apolipoprotein B, HDL-cholesterol, LDL-cholesterol and Triglycerides.

Table S6. Multivariable MR model 2 estimates for Apolipoprotein A-I, HDL-cholesterol, Triglycerides and Body mass index.

Table S7. Selection of Instrument Variables for lipids and apolipoproteins.

**Supplementary Figures.**

Figure S1. Scatter plots of lipids and apolipoproteins:(A)Apolipoprotein A-I, (B)Apolipoprotein B, (C)HDL-cholesterol, (D)LDL-cholesterol, (E)Triglycerides.

Figure S2. Funnel plot of lipids and apolipoproteins:(A)Apolipoprotein A-I, (B)Apolipoprotein B, (C)HDL-cholesterol, (D)LDL-cholesterol, (E)Triglycerides.

Table S1. Additional information on GWAS, including covariates adjusted for.

| **Exposure** | **Case/control** | **Population** | **Covariates adjusted for** | **Pubmed ID/GWAS** |
| --- | --- | --- | --- | --- |
| Apolipoprotein A-I | 393,193 | Predominantly European, with additional subsets from East Asian ancestries | Age, sex, recruitment centre | 32203549 |
| Apolipoprotein B | 393,193 | Predominantly European, with additional subsets from East Asian ancestries | Age, sex, recruitment centre | 32203549 |
| LDL-cholesterol | 343,992 | European | Age, sex, principal components | 34594039 |
| HDL-cholesterol | 187,167 | Predominantly European, with additional subsets from East Asian, South Asian, and African ancestries | Age, sex, principal components. Individuals known to be on lipid-lowering medications were excluded | 24097068 |
| Triglycerides | 343,992 | European | Age, sex, principal components | 34594039 |
| GDM | 5,687 / 117,892 | European | Age, genotyping batch, 10 principal components | finn-b-GEST_DIABETES |

LDL = low-density lipoprotein, HDL = high density lipoprotein

**Table S2. Cochran Q test result and MR-Egger intercept in univariable MR**

| **Exposure** | **IVW-Q test** | | **MR-Egger** | | |
| --- | --- | --- | --- | --- | --- |
|  | **Q-statistic** | **Q_*P* value** | **Intercept** | **SE** | ***P* value** |
| Apolipoprotein A-I | 165.16 | 8.49 x 10^-2^ | 2.33 x 10^-2^ | 1.91 x 10^-2^ | 2.51 x 10^-1^ |
| Apolipoprotein B | 280.12 | 1.57 x 10^-6^ * | 2.10 x 10^-3^ | 3.30 x 10^-3^ | 5.25 x 10^-1^ |
| LDL-cholesterol | 205.11 | 1.92 x 10^-1^ | -2.73 x 10^-3^ | 3.34 x 10^-3^ | 4.13 x 10^-1^ |
| HDL-cholesterol | 114.17 | 2.16 x 10^-2^ * | -2.01 x 10^-3^ | 1.64 x 10^-3^ | 2.53x 10^-1^ |
| Triglycerides | 219.25 | 7.92 x 10^-2^ | -1.83 x 10^-3^ | 3.12 x 10^-3^ | 6.87x 10^-1^ |

**Table S3. Results of univariable MR.**

| **Exposure** | **Method** | **OR(95% CI)** | ***P* value** |
| --- | --- | --- | --- |
| Apolipoprotein A-I | IVW | 0.76 (0.68 to 0.86) | 1.49 x 10^-5^ * |
|  | Weighted median | 0.79 (0.67 to 0.94) | 7.65 x 10^-3^ * |
|  | Weighted mode | 0.89 (0.75 to 1.05) | 1.97 x 10^-1^ |
|  | MR Egger | 0.94 (0.78 to 1.13) | 5.43 x 10^-1^ |
| Apolipoprotein B | IVW | 1.04 (0.92 to 1.17) | 5.14x 10^-1^ |
|  | Weighted median | 0.99 (0.83 to 1.19) | 9.64 x 10^-1^ |
|  | Weighted mode | 1.06 (0.93 to 1.22) | 3.39 x 10^-1^ |
|  | MR Egger | 1.07 (0.91 to 1.26) | 3.64 x 10^-1^ |
| LDL-cholesterol | IVW | 0.98 (0.87 to 1.10) | 7.47 x 10^-1^ |
|  | Weighted median | 1.01 (0.83 to 1.21) | 9.15 x 10^-1^ |
|  | Weighted mode | 1.00 (0.88 to 1.15) | 8.89 x 10^-1^ |
|  | MR Egger | 1.02 (0.87 to 1.19) | 7.80 x 10^-1^ |
| HDL-cholesterol | IVW | 0.79 (0.69 to 0.89) | 2.73x 10^-4^ * |
|  | Weighted median | 0.84 (0.69 to 1.01) | 6.90 x 10^-2^ |
|  | Weighted mode | 0.90 (0.75 to 1.07) | 2.63 x 10^-1^ |
|  | MR Egger | 1.05 (0.83 to 1.32) | 6.78 x 10^-1^ |
| Triglycerides | IVW | 1.28 (1.12 to 1.46) | 2.38 x 10^-4^ * |
|  | Weighted median | 1.16 (0.95 to 1.41) | 1.39 x 10^-1^ |
|  | Weighted mode | 1.27 (1.06 to 1.52) | 7.56 x 10^-3^ * |
|  | MR Egger | 1.06 (0.87 to 1.29) | 5.24 x 10^-1^ |

LDL = low-density lipoprotein, HDL = high density lipoprotein, IVW=Inverse variance weighted

**Table S4. Effects of genetic liability to gestational diabetes mellitus on lipids and apolipoproteins in reverse Mendelian randomization analysis**

| **Exposure** | **Outcome** | **Method** | **OR(95% CI)** | ***P* value** |
| --- | --- | --- | --- | --- |
| Gestational diabetes mellitus | Apolipoprotein A-I | IVW | 1.00 (0.97 to 1.04) | 6.46 x 10^-1^ |
|  |  | Weighted median | 0.99 (0.98 to 1.00) | 1.50 x 10^-1^ |
|  |  | Weighted mode | 0.99 (0.98 to 1.00) | 2.36 x 10^-1^ |
|  |  | MR Egger | 0.94 (0.81 to 1.08) | 4.71 x 10^-1^ |
|  | Apolipoprotein B | IVW | 0.97 (0.95 to 1.00) | 6.57x 10^-1^ |
|  |  | Weighted median | 0.98 (0.97 to 0.99) | 2.06 x 10^-2^ * |
|  |  | Weighted mode | 0.99 (0.97 to 1.00) | 2.54 x 10^-1^ |
|  |  | MR Egger | 1.05 (0.98 to 1.32) | 2.22 x 10^-1^ |
|  | LDL-cholesterol | IVW | 0.95 (0.90 to 1.01) | 1.51 x 10^-1^ |
|  |  | Weighted median | 0.98 (0.96 to 0.99) | 1.50 x 10^-2^ * |
|  |  | Weighted mode | 0.99 (0.97 to 1.00) | 4.56 x 10^-1^ |
|  |  | MR Egger | 1.12 (1.07 to 1.16) | 4.62 x 10^-3^ * |
|  | HDL-cholesterol | IVW | 1.01 (0.97 to 1.05) | 3.85 x 10^-1^ |
|  |  | Weighted median | 1.01 (0.99 to 1.03) | 1.15 x 10^-1^ |
|  |  | Weighted mode | 1.01 (0.99 to 1.03) | 2.65 x 10^-1^ |
|  |  | MR Egger | 0.96 (0.89 to 1.05) | 5.22 x 10^-1^ |
|  | Triglycerides | IVW | 0.95 (0.78 to 1.16) | 6.61 x 10^-1^ |
|  |  | Weighted median | 1.02 (1.01 to 1.03) | 6.06 x 10^-5^ * |
|  |  | Weighted mode | 1.02 (1.01 to 1.03) | 8.35 x 10^-3^ * |
|  |  | MR Egger | 1.02 (1.00 to 1.03) | 6.05 x 10^-2^ |

LDL = low-density lipoprotein, HDL = high density lipoprotein, IVW=Inverse variance weighted

**Table S5. Multivariable MR model 1 estimates for lipids and apolipoproteins.**

| **Exposure** | **SNPs** | **beta** | **Se** | **OR(95% CI)** | ***P* value** |
| --- | --- | --- | --- | --- | --- |
| Apolipoprotein A-I | 408 | -0.52 | 0.22 | 0.59 (0.38 to 0.91) | 1.84 x 10^-2^ * |
| Apolipoprotein B | 408 | -0.14 | 0.34 | 0.86 (0.43 to 1.69) | 6.65 x 10^-1^ |
| LDL-cholesterol | 408 | 0.05 | 0.35 | 1.06 (0.52 to 2.13) | 8.67 x 10^-1^ |
| HDL-cholesterol | 408 | 0.25 | 0.23 | 1.28 (0.81 to 2.02) | 2.74 x 10^-1^ |
| Triglycerides | 408 | 0.17 | 0.11 | 1.19 (0.95 to 1.49) | 1.25 x 10^-1^ |

**Table S6. Multivariable MR model 2 estimates for Apolipoprotein A-I, HDL-cholesterol, Triglycerides and Body mass index.**

| **Exposure** | **SNPs** | **beta** | **Se** | **OR(95% CI)** | ***P* value** |
| --- | --- | --- | --- | --- | --- |
| Apolipoprotein A-I | 404 | -0.52 | 0.22 | 0.59 (0.38 to 0.92) | 1.96 x 10^-2^ * |
| HDL-cholesterol | 404 | 0.28 | 0.23 | 1.33 (0.85 to 2.07) | 2.09 x 10^-1^ |
| Triglycerides | 404 | 0.12 | 0.10 | 1.13 (0.92 to 1.37) | 2.24 x 10^-1^ |
| Body mass index | 404 | 0.45 | 0.10 | 1.58 (1.29 to 1.93) | 6.93 x 10^-6^ * |

**Table S7. Selection of Instrument Variables for lipids and apolipoproteins.**

| **Exposure** | **Outcome** | **SNPs** | **Median of F*** | **Minimum of F** | **Maximum of F** |
| --- | --- | --- | --- | --- | --- |
| Apolipoprotein A-I | GDM | 261 | 56.58 | 23.49 | 9322.07 |
| Apolipoprotein B | GDM | 179 | 54.37 | 20.73 | 29776.66 |
| LDL-cholesterol | GDM | 147 | 59.51 | 28.60 | 14220.48 |
| HDL-cholesterol | GDM | 86 | 56.54 | 29.94 | 1674.15 |
| Triglycerides | GDM | 216 | 55.89 | 29.08 | 6876.67 |

**^*^**An F statistic greater than 10 is generally considered strong enough to reduce bias due to weak instrumental variables.

**Figure S1. Scatter plots of lipids and apolipoproteins:(A)Apolipoprotein A-I, (B)Apolipoprotein B, (C)HDL-cholesterol, (D)LDL-cholesterol, (E)Triglycerides**

A B


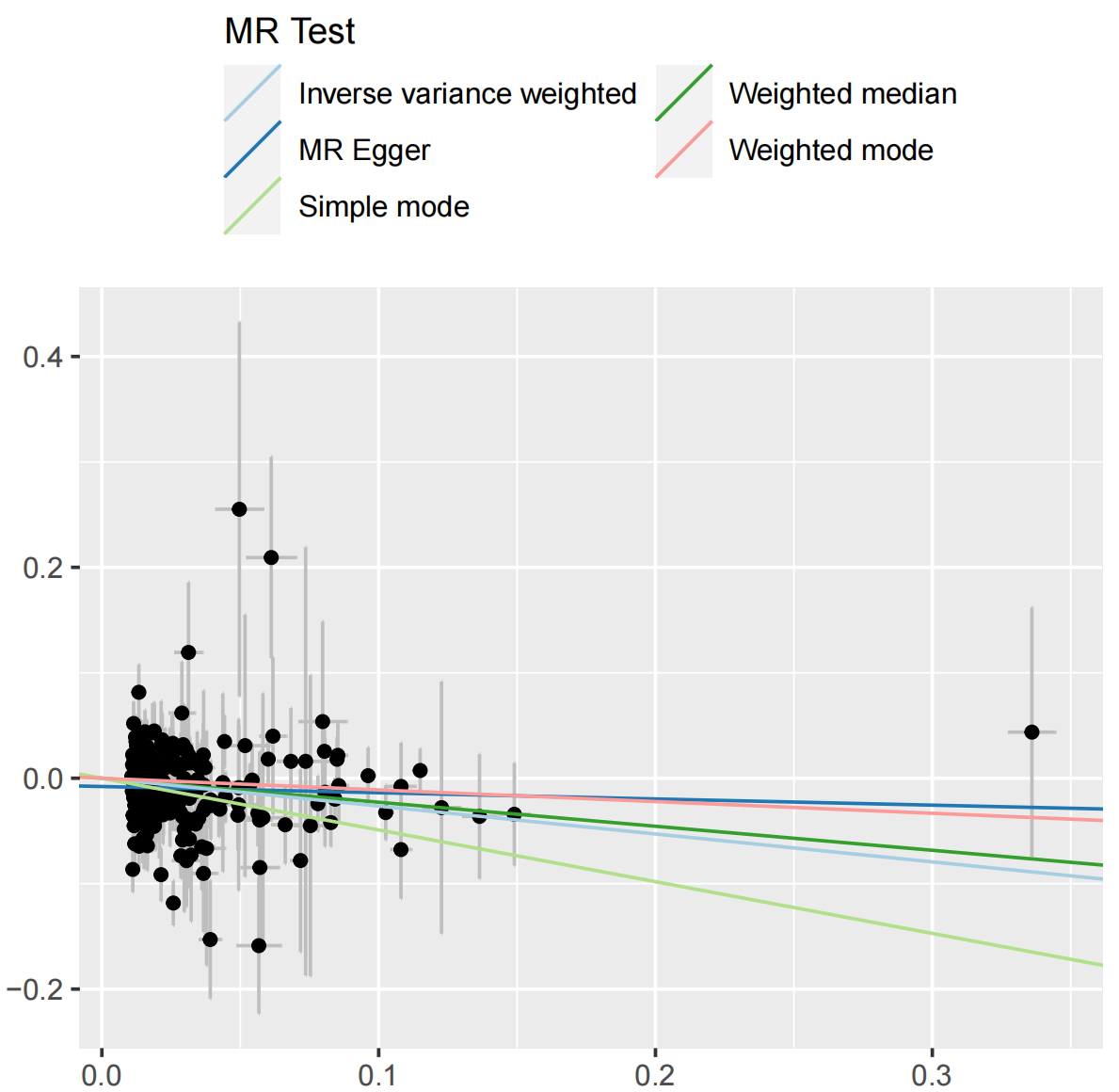

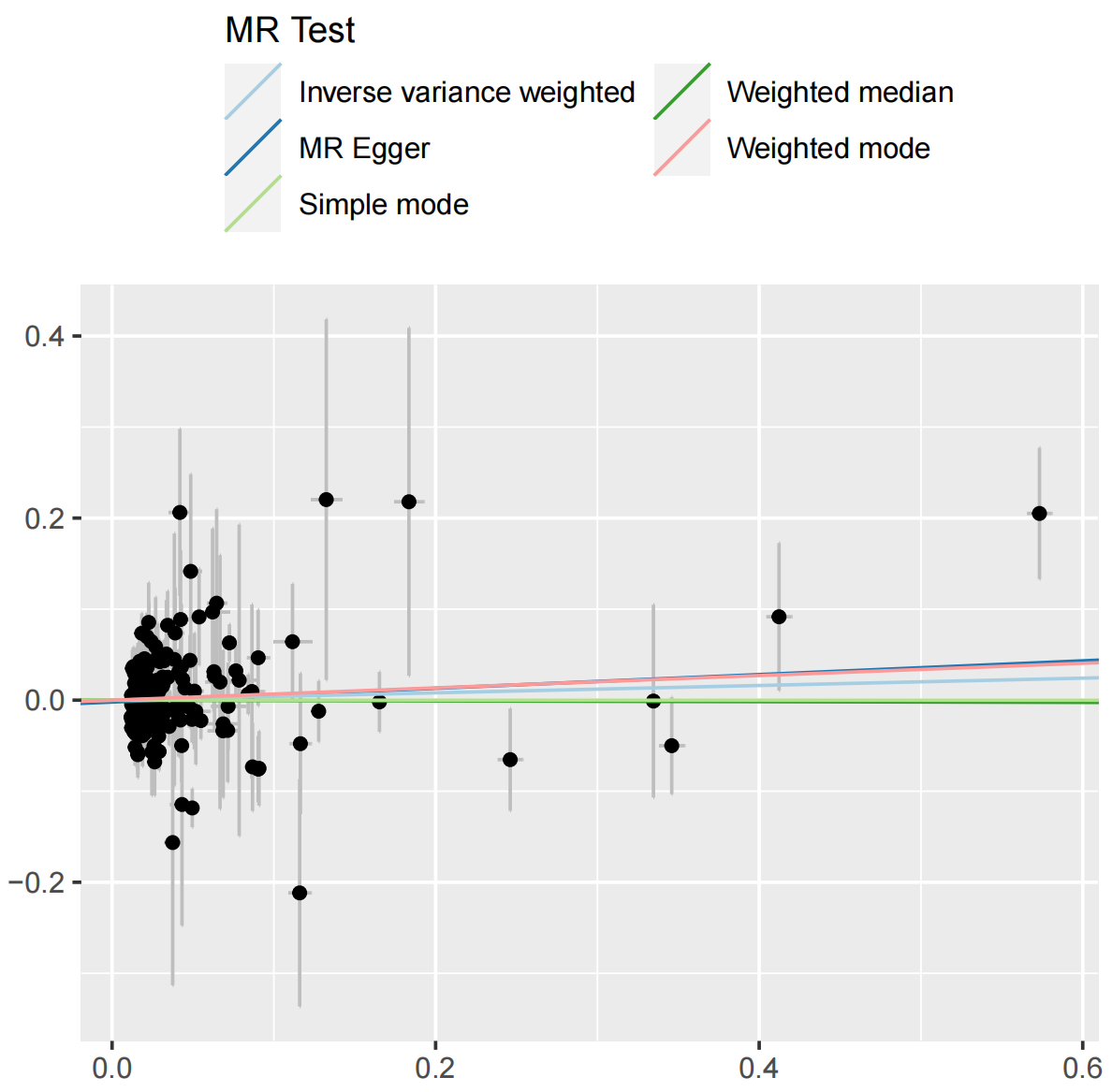


C D


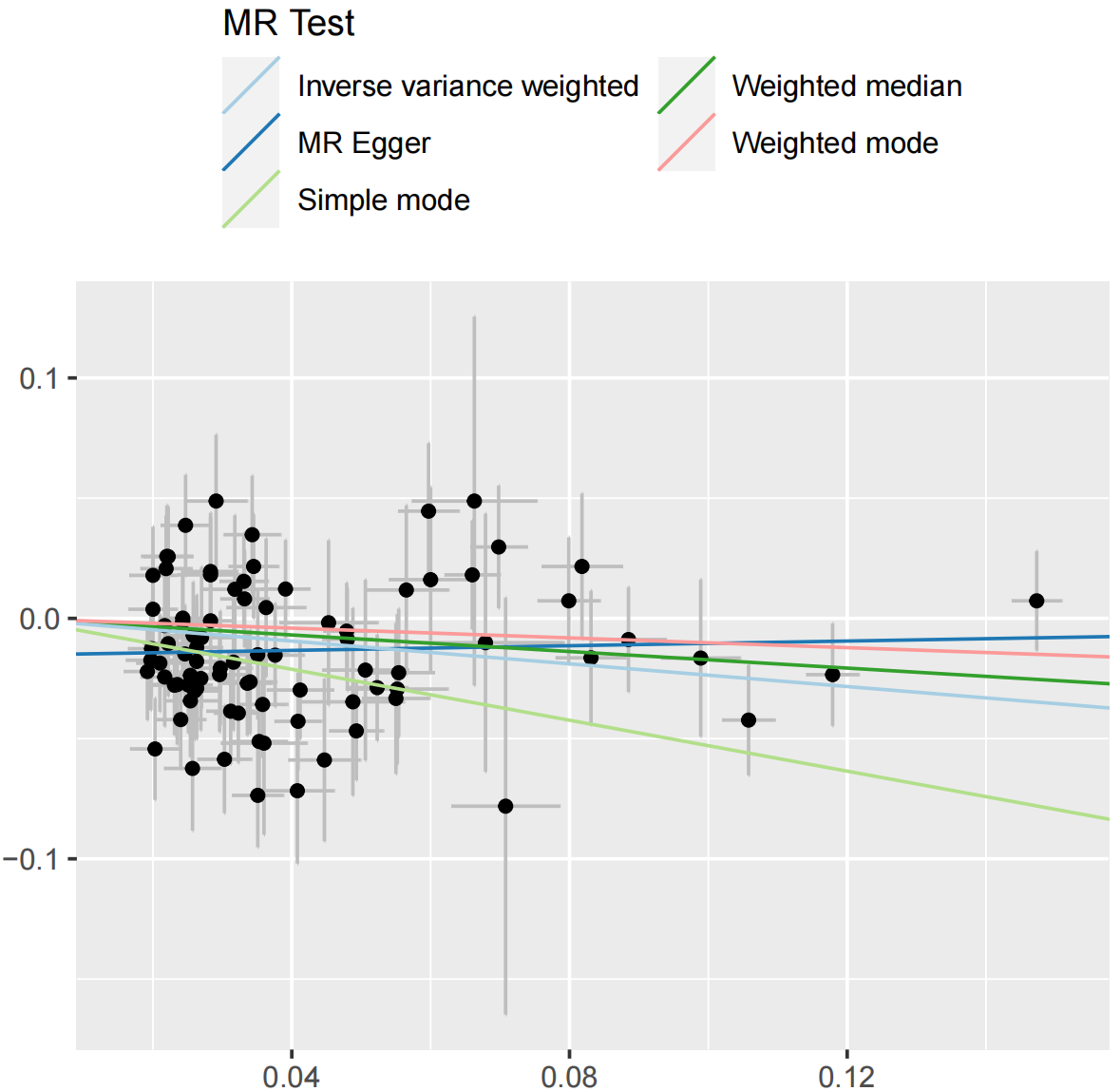

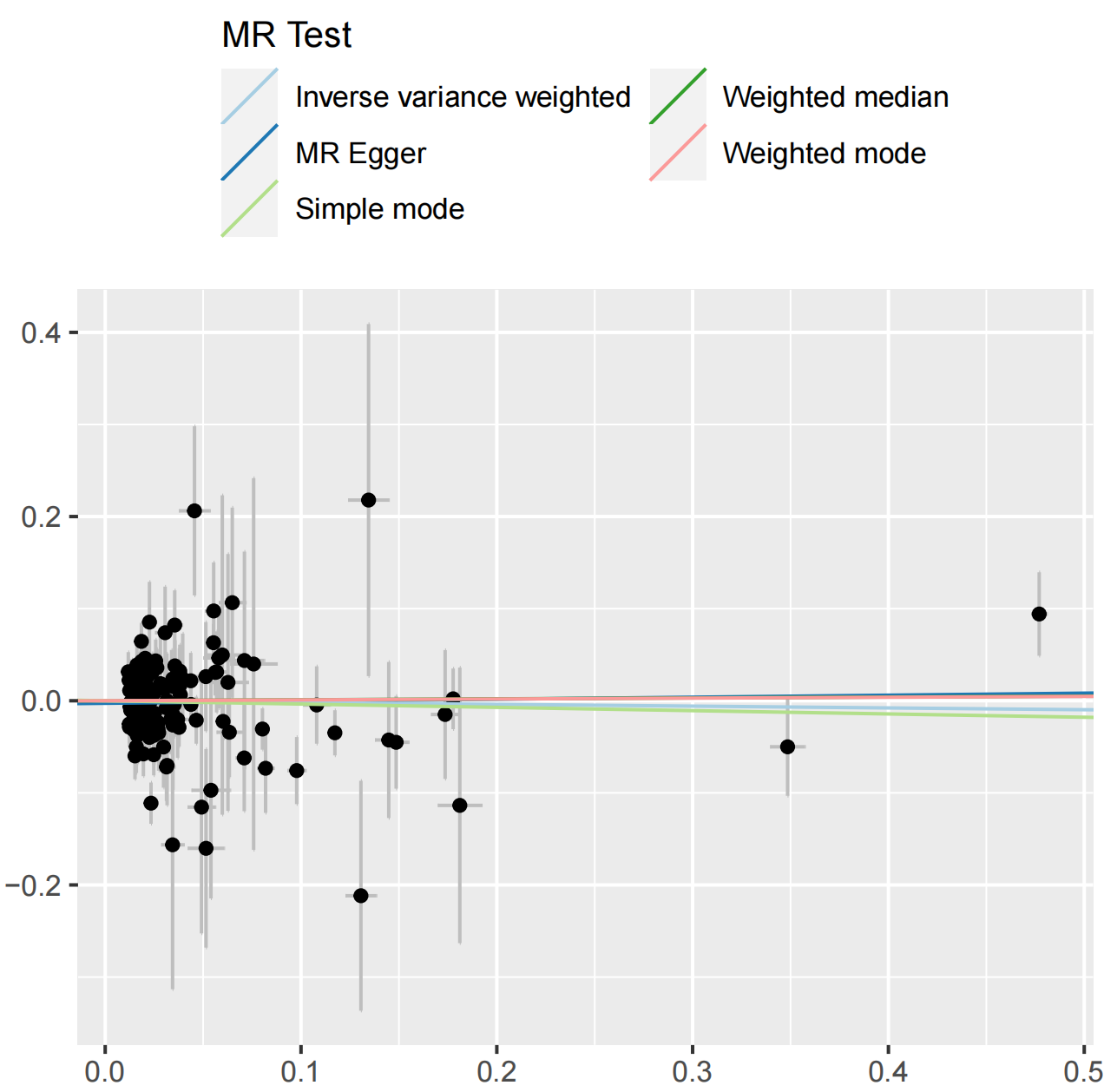


E


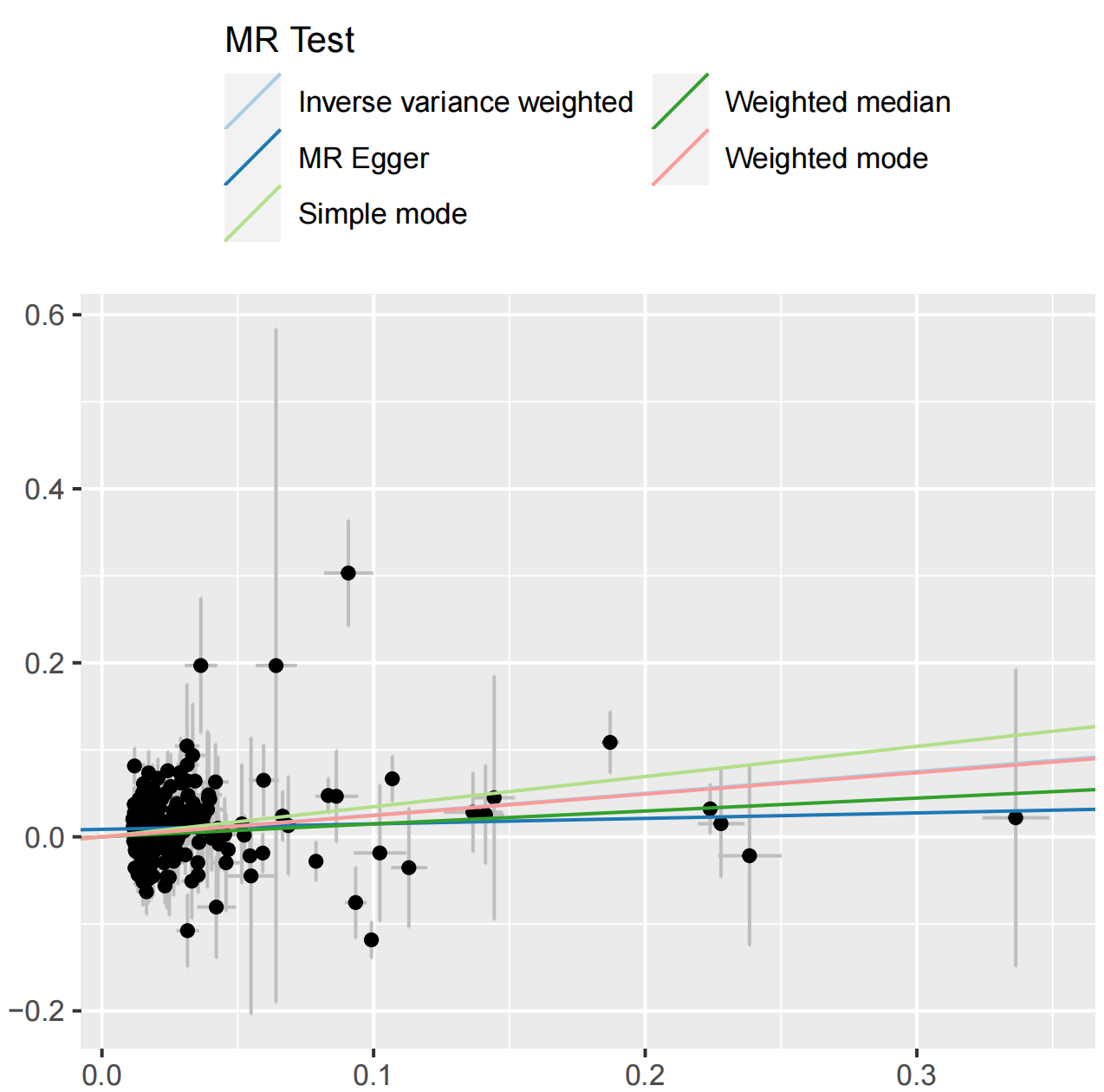


**Figure S2. Funnel plot of lipids and apolipoproteins:(A)Apolipoprotein A-I, (B)Apolipoprotein B, (C)HDL-cholesterol, (D)LDL-cholesterol, (E)Triglycerides**

A B


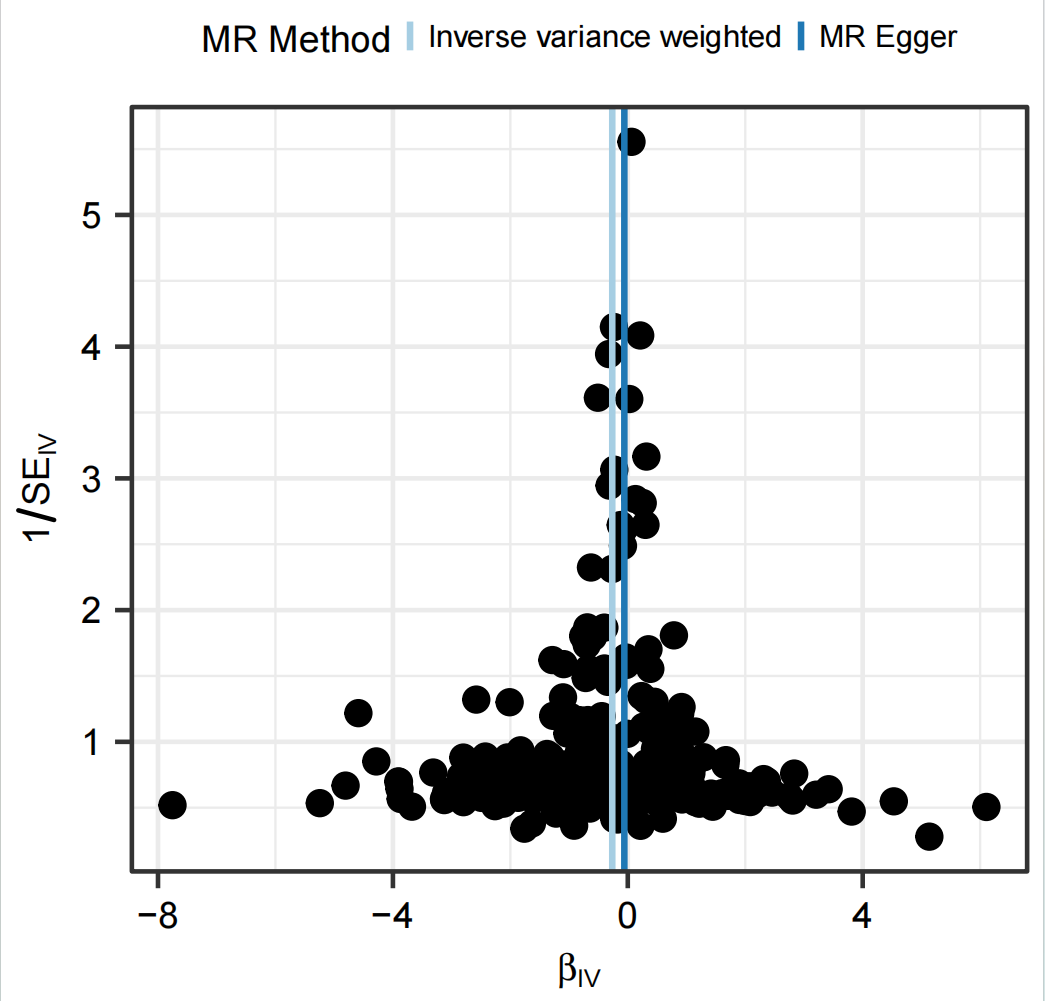

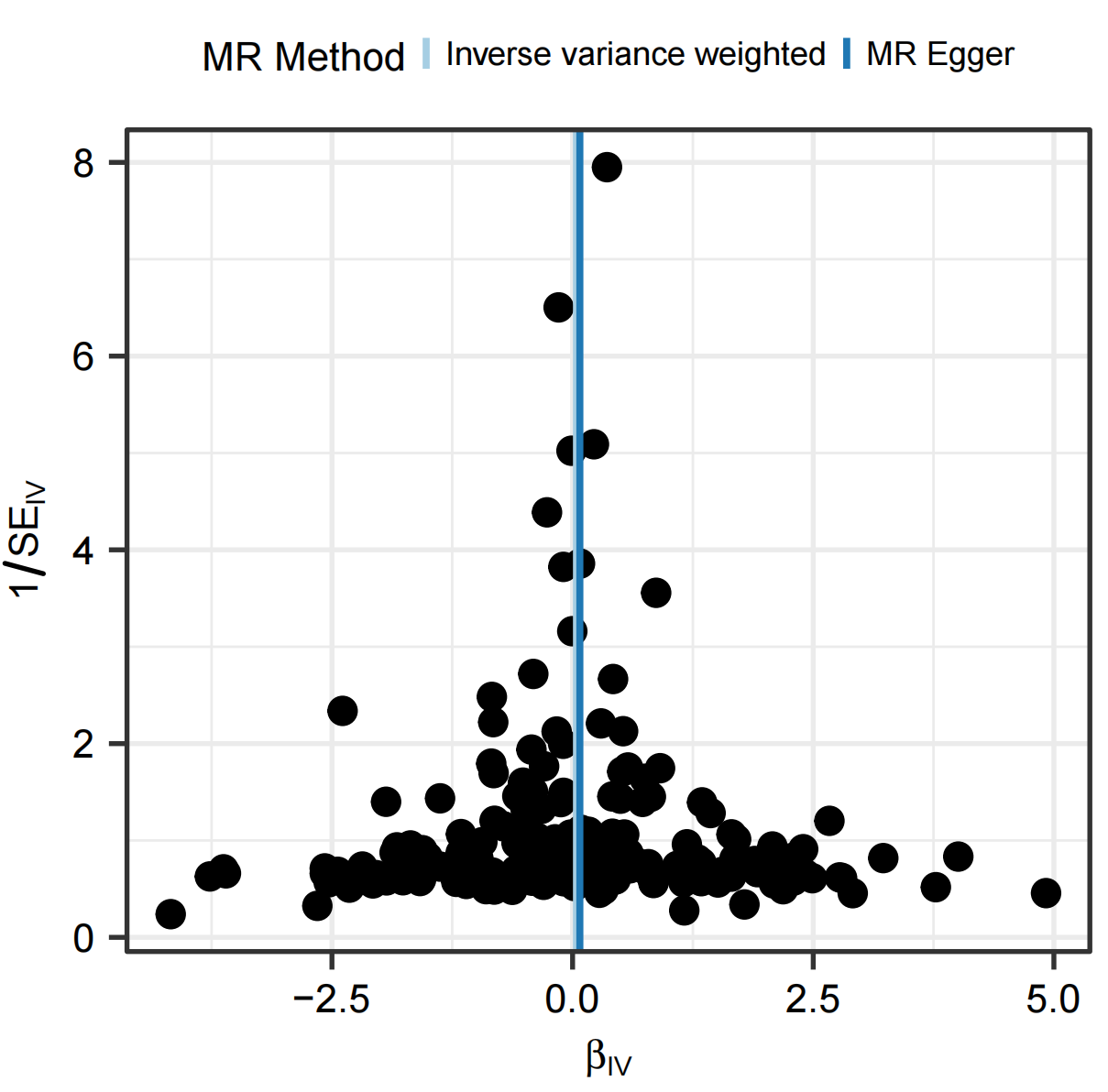


C D


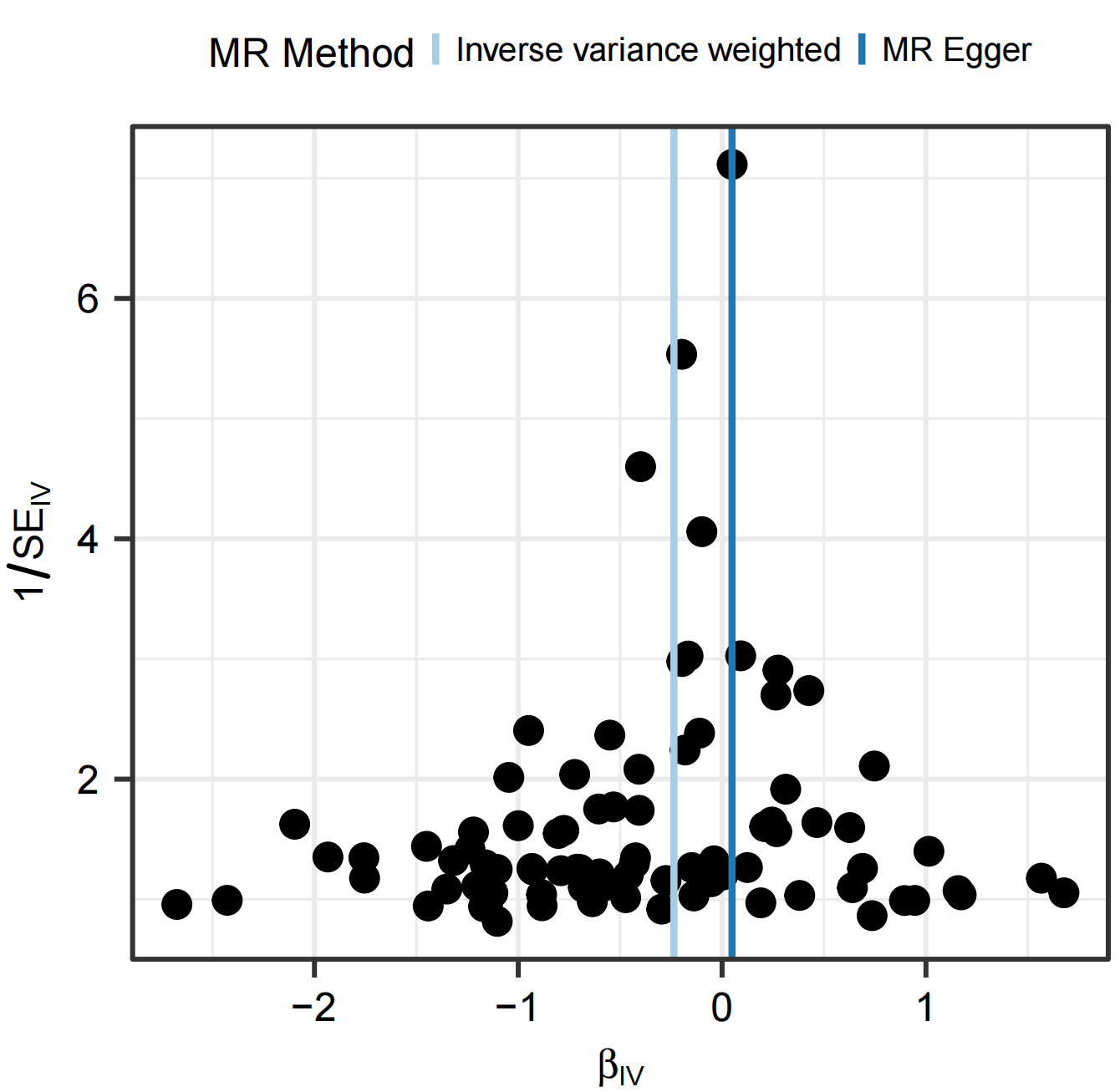

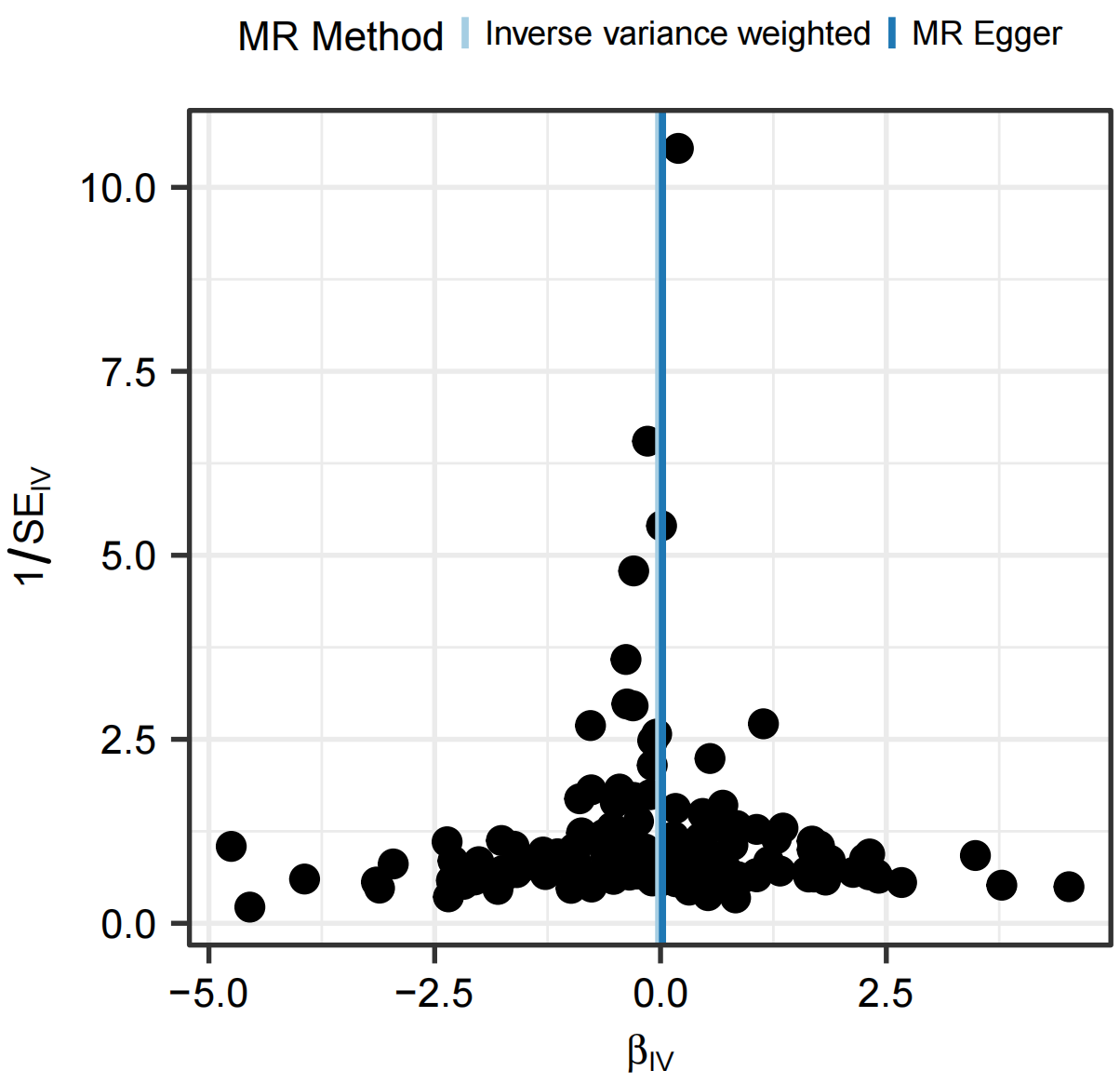


E


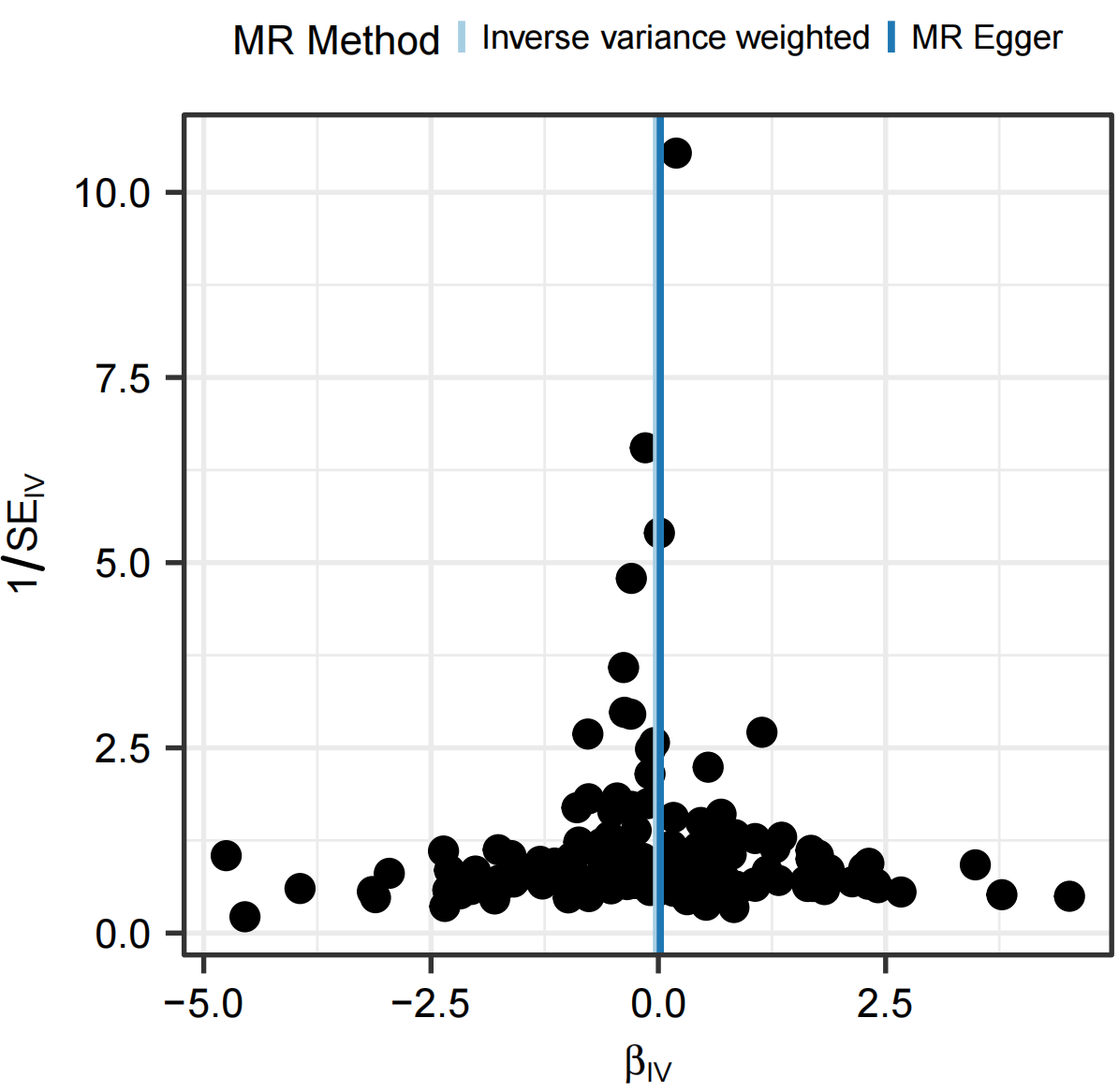

Supplement: Supplementary file 1 — Supplementary Material 1. [file 12884_2024_6556_MOESM1_ESM.doc]
